# Bronchial angiolipoma successfully treated by sleeve resection of the right bronchus intermedius: a case report

**DOI:** 10.1186/s12893-019-0481-0

**Published:** 2019-01-31

**Authors:** Satoshi Kamata, Itaru Ishida, Takafumi Sugawara, Hiroshi Yaegashi, Hiroyuki Oura

**Affiliations:** 1 Department of Thoracic Surgery, Iwate Prefectural Central Hospital, Ueda 1-4-1, Morioka, 020-0066 Japan; 2 Department of Pathology, Iwate Prefectural Central Hospital, Ueda 1-4-1, Morioka, Japan

**Keywords:** Angiolipoma, Lung, Bronchial sleeve resection

## Abstract

**Background:**

Angiolipoma is a rare, benign tumor that primarily develops in the limbs and trunk. The occurrence of angiolipoma in the lungs is extremely rare; to date, only two cases of primary bronchial angiolipoma have been reported. Here, we report a case of angiolipoma of the right bronchus intermedius that was successfully treated with sleeve resection and reconstructive surgery.

**Case presentation:**

This report presents a case of angiolipoma that developed in the right bronchus intermedius of a 68-year-old man. A chest CT revealed a 10-mm endobronchial mass that was clearly visible as a high-attenuation area of contrast enhancement. Bronchoscopy revealed a submucosal tumor on the anterior wall of the entrance to the right bronchus intermedius that was constricting the airway lumen. The tumor surface was covered with numerous engorged blood vessels, and the middle and inferior pulmonary lobes were intact. Bronchial sleeve resection of the right bronchus intermedius was performed. Histologically, a mixture of proliferating blood vessels and adipocytes were observed within the bronchus wall. Therefore, the pathological diagnosis was angiolipoma. Lung function was preserved, and complete resection of the tumor was achieved. At present (2 years and 7 months after surgery), the patient is recurrence-free.

**Conclusion:**

Accordingly, using bronchial sleeve resection and end-to-end anastomosis techniques, we accomplished complete tumor excision and avoided the need to resect additional lung parenchyma. Our procedure preserved pulmonary function and yielded a curative result. Bronchoscopic intervention or minimal parenchymal resection should be considered as treatments for bronchial angiolipoma. Given the small number of reports of bronchial angiolipoma, the collection of additional data is important to elucidate the clinical characteristics of this rare tumor.

## Background

To the best of our knowledge, only two cases of angiolipoma that developed in the lung have been reported [1, 2]. Here, we report a case of angiolipoma of the right bronchus intermedius that was successfully treated with sleeve resection and reconstructive surgery.

## Case presentation

**Fig. 1:**
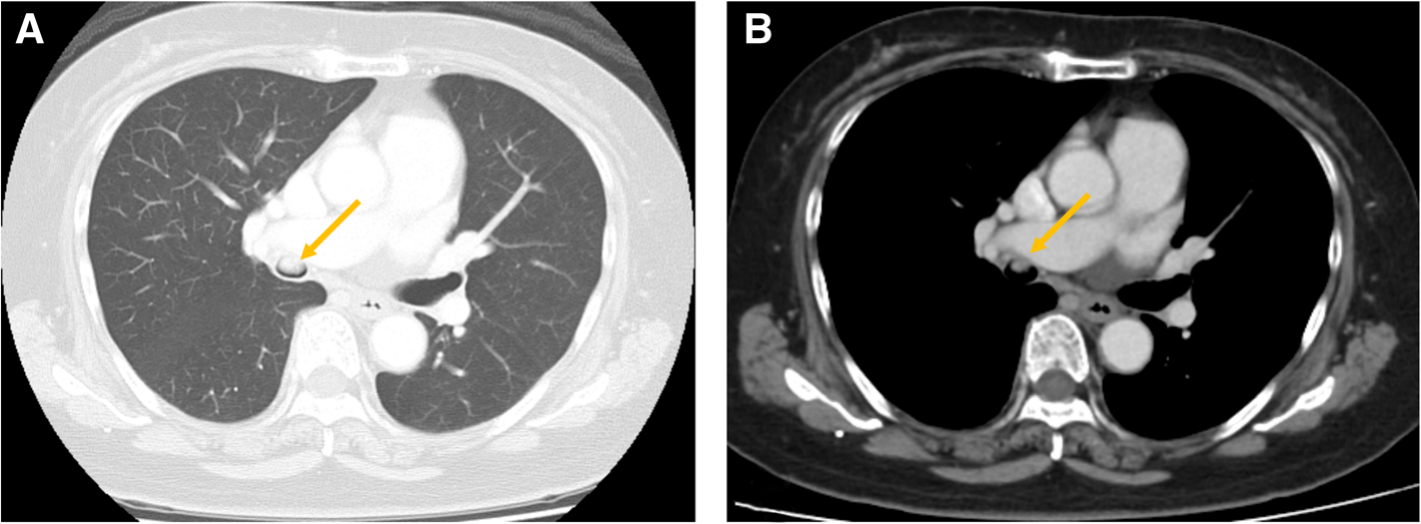
A protruding mass is observed within the right bronchus intermedius (arrow). **a** Lung window. **b** Mediastinal window

**Fig. 2:**
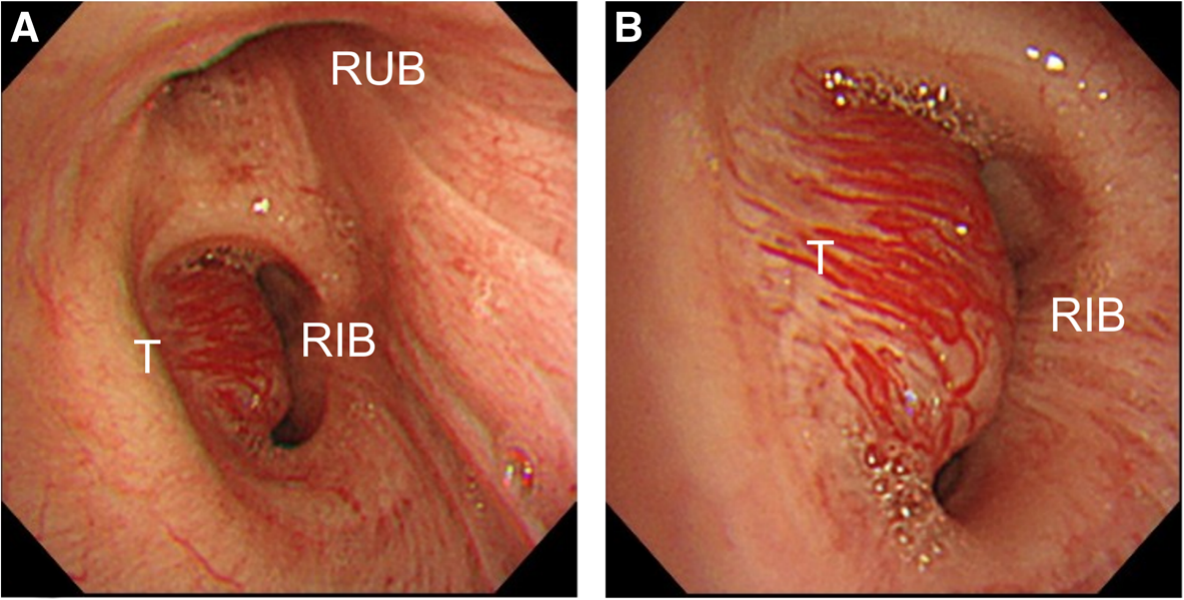
**a** Bronchoscopy reveals a submucosal tumor at the entrance of the right bronchus intermedius. The tumor is arising from the anterior wall of the airway, resulting in stenosis. **b** The tumor is covered with numerous engorged blood vessels. RIB: right intermediate bronchus; RUB: right upper bronchus; T: tumor

A 68-year-old man was referred to our department with infraclavicular lymphadenopathy and an endobronchial tumor that was incidentally discovered on computed tomography (CT) at another hospital. An excision of the left infraclavicular nodes was performed, but no malignant findings were observed. A chest CT revealed a 10-mm endobronchial mass that was clearly visible as a high-attenuation area of contrast enhancement (Fig. 1a, b). An 18-fluorodeoxyglucose positron emission tomographic whole-body scan revealed no significant uptake in the lesion. Bronchoscopy revealed a submucosal tumor on the anterior wall of the entrance to the right bronchus intermedius that was constricting the airway lumen (Fig. 2a, b). The tumor surface was covered with numerous engorged blood vessels, and the middle and inferior pulmonary lobes were intact. Although a biopsy of the mass was performed, no definitive diagnosis was achieved.

A posterolateral thoracotomy was performed through the fifth intercostal space under general anesthesia. The bronchus intermedius was dissected, and the membranous portion was opened to expose the lumen. The distal end of the tumor was transected first followed by the proximal end, providing adequate tumor-free margins. Because the tumor had clearly defined borders, the resection line was determined by macroscopically securing the margin from the tumor. Subsequently, the tumor and bronchus intermedius were removed en bloc. The tumor measured 13 × 6 mm in size and was hemispherical in morphology. Examination of frozen tumor sections suggested angioma with no malignant findings. The presence of tumor-free margins at both the proximal and distal ends of the bronchus was also confirmed by examination of frozen sections. The excised segment of the bronchus measured 1 cm in length; thus, the bronchus was reconstructed by end-to-end anastomosis using 3–0 PDS (polydioxanone) sutures without excessive tension. The anastomosis was then wrapped in a pedicled intercostal muscle flap to isolate it from the pulmonary artery.

**Fig. 3:**
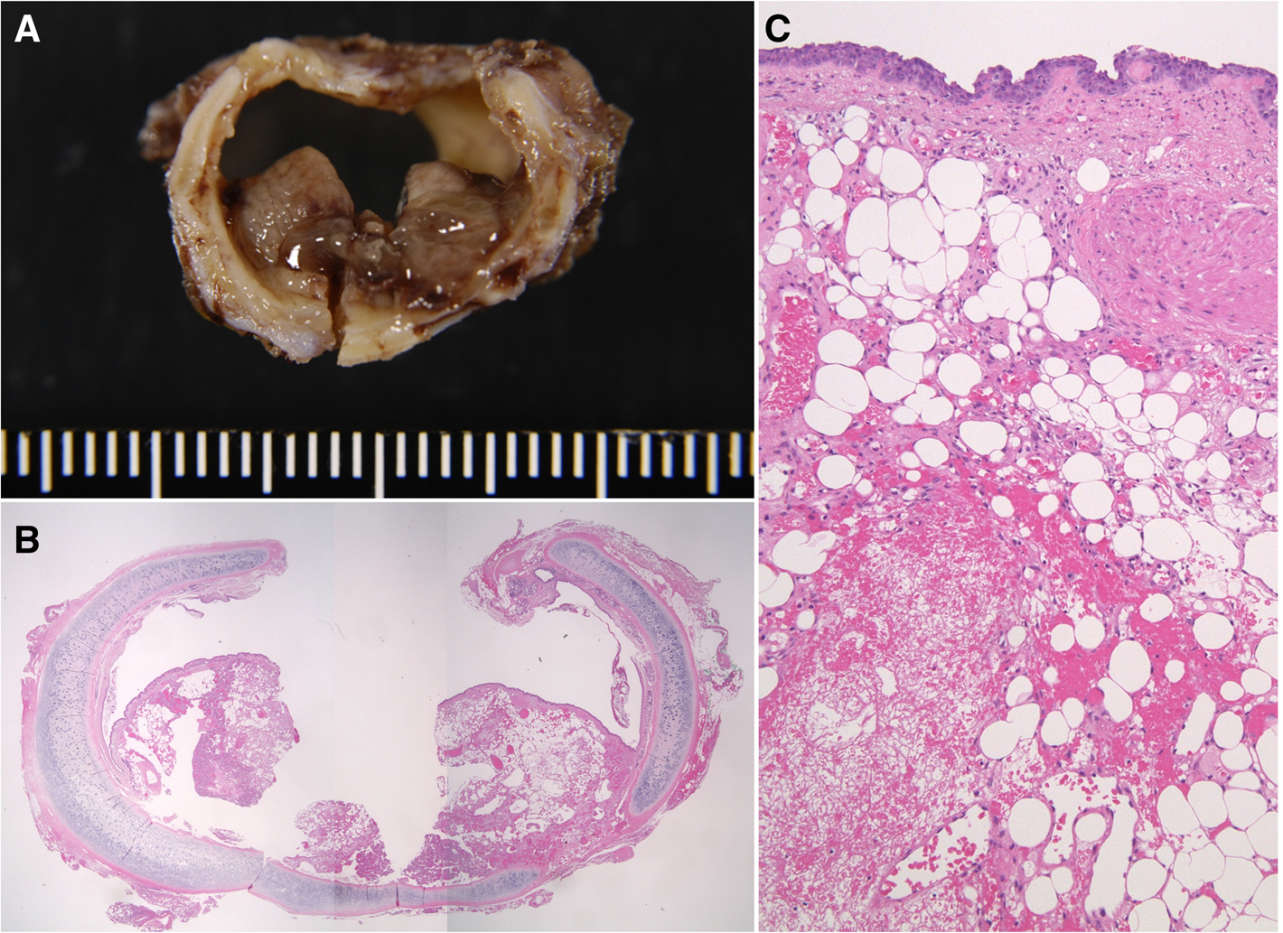
**a** Formalin-fixed resected specimen. The surface of this hemispherical lesion was covered with normal bronchial mucosa. **b** A histological image of the specimen demonstrates an endobronchial tumor. Hematoxylin and eosin stain; original magnification: × 12.5. **c** The surface of the tumor was covered with normal bronchial epithelium, and was composed of adipose tissue and numerous blood vessels. Hematoxylin and eosin stain; original magnification: × 200

Histologically, a mixture of proliferating blood vessels and adipocytes were observed within the bronchus wall (Fig. 3a–c). Therefore, the pathological diagnosis was angiolipoma. The patient experienced no postoperative complications and was discharged on postoperative day 15. Two years and 7 months postsurgery, the patient has experienced no recurrence.

## Discussion and conclusions

Angiolipoma is a rare benign neoplasm. The first reported case of angiolipoma, which was described by Bowen in 1912 [3], was in a patient with subcutaneous lesions in all four limbs. The most common sites of angiolipoma occurrence include the upper and lower limbs, abdomen, precordium, and back. In contrast, the occurrence of angiolipoma in the lung is extremely rare, and only two cases have been reported to date [1, 2]. Similar to the present case, the tumor developed in the right bronchus intermedius in one of these cases. In the other case, the tumor developed in the bronchus of the right inferior lobe [1, 2]. In each of these cases, angiolipoma developed in the central bronchus and protruded into the airway. Due to the indolent nature of this neoplasm, symptoms of bronchial obstruction, such as coughing, wheezing, dyspnea, sputum production, hemoptysis, atelectasis, and pneumonia, may be observed in these patients.

Histologically, angiolipoma consists of mature adipose and vascular tissues in varying proportions [3, 4]. The vascular tissue is predominantly located in the tumor periphery and may include capillaries with an occasional fibrin thrombus. In the present case, we observed a mixture of proliferating capillaries and small blood vessels as well as proliferating adipocytes between the mucosal epithelium and bronchial cartilage. In addition, the tumor surface was lined with normal bronchial epithelium. This finding suggests that endobronchial angiolipoma arises in bronchial submucosal adipose tissue, similarly to endobronchial lipoma. Angiolipomas are histologically classified into two types: infiltrating and non-infiltrating. Infiltrating angiolipomas are not capsulated and have the tendency to invade and spread to the surrounding tissues [5]. The present case did not exhibit such a tendency and was, therefore, considered to be a non-infiltrating angiolipoma.

With respect to treatment, bronchoscopic intervention should be considered as the first choice of therapy for benign endobronchial tumors. Previously reported cases of angiolipoma have been treated with either (a) surgical excision or (b) bronchoscopic resection with a high-frequency electric snare together with argon plasma coagulation under general anesthesia. Due to the risk of recurrence, the authors of the former case study recommended surgical resection for patients in whom surgery could be tolerated [1]. In the latter case, the authors preferred a bronchoscopic approach due to the low risk of malignant transformation of this rare, benign tumor [2]. In the present case, the lesion was located in the right bronchus intermedius and was covered with numerous engorged blood vessels. We decided to perform open surgery rather than endoscopic resection because (a) the endoscopic resection procedure may increase the risk of tumor bleeding; (b) complete resection with a high-frequency electric snare is potentially challenging due to the hemispherical, rather than polypoid, tumor morphology; (c) the presence of a malignant tumor (e.g., a carcinoid tumor) should not be discounted; and (d) bronchial sleeve resection and reconstruction could be performed safely given that the length of the longitudinal tumor axis was relatively short. Notably, a lung-sparing technique should be considered when benign tumors are treated surgically. Accordingly, using bronchial sleeve resection and end-to-end anastomosis techniques, we accomplished complete tumor excision and avoided the need to resect additional lung parenchyma. Our procedure preserved pulmonary function and yielded a curative result. As a treatment option for angiolipomas other than surgical resection or bronchoscopic intervention, radiotherapy can be considered. In case of incomplete resection for infiltrating angiolipoma, postoperative radiotherapy should be recommended. However, in the present case, because complete resection was achieved, postoperative irradiation was not performed.

In conclusion, bronchoscopic intervention or minimal parenchymal resection should be considered as treatments for bronchial angiolipoma. Given the small number of reports of bronchial angiolipoma, the collection of additional data is important to elucidate the clinical characteristics of this rare tumor.

## Abbreviations

CT: Computed tomography
